# Organic and Inorganic PCL-Based Electrospun Fibers

**DOI:** 10.3390/polym12061325

**Published:** 2020-06-10

**Authors:** Adrián Leonés, Alicia Mujica-Garcia, Marina Patricia Arrieta, Valentina Salaris, Daniel Lopez, José Maria Kenny, Laura Peponi

**Affiliations:** 1 Instituto de Ciencia y Tecnología de Polímeros (ICTP-CSIC), C/Juan de la Cierva 3, 28006 Madrid, Spain; aleones@ictp.csic.es (A.L.); alicia.mujica@unipg.it (A.M.-G.); marrie06@ucm.es (M.P.A.); valen.salaris@gmail.com (V.S.); daniel.l.g@csic.es (D.L.); jose.kenny@unipg.it (J.M.K.); 2 Interdisciplinary Platform for Sustainable Plastics towards a Circular Economy—The Spanish National Research Council (SusPlast-CSIC), 28006 Madrid, Spain; 3 Facultad de Óptica y Optometría, Universidad Complutense de Madrid (UCM), Arcos de Jalón 118, 28037 Madrid, Spain; 4 Civil and Environmental Engineering Department, University of Perugia, Via G, Duranti 93, 06125 Perugia, Italy

**Keywords:** electrospinning, PCL, organic nanoparticles, inorganic nanoparticles

## Abstract

In this work, different nanocomposite electrospun fiber mats were obtained based on poly(e-caprolactone) (PCL) and reinforced with both organic and inorganic nanoparticles. In particular, on one side, cellulose nanocrystals (CNC) were synthesized and functionalized by “*grafting from*” reaction, using their superficial OH– group to graft PCL chains. On the other side, commercial chitosan, graphene as organic, while silver, hydroxyapatite, and fumed silica nanoparticles were used as inorganic reinforcements. All the nanoparticles were added at 1 wt% with respect to the PCL polymeric matrix in order to compare the different behavior of the woven no-woven nanocomposite electrospun fibers with a fixed amount of both organic and inorganic nanoparticles. From the thermal point of view, no difference was found between the effect of the addition of organic or inorganic nanoparticles, with no significant variation in the T_g_ (glass transition temperature), T_m_ (melting temperature), and the degree of crystallinity, leading in all cases to high crystallinity electrospun mats. From the mechanical point of view, the highest values of Young modulus were obtained when graphene, CNC, and silver nanoparticles were added to the PCL electrospun fibers. Moreover, all the nanoparticles used, both organic and inorganic, increased the flexibility of the electrospun mats, increasing their elongation at break.

## 1. Introduction

Poly(ε-caprolactone) (PCL) is a biodegradable and biocompatible aliphatic polyester. It offers a unique combination of polyolefin-like mechanical properties and polyester-like hydrolyzability [1,2], providing good compatibility with a wide range of other polymers in both blends and copolymer forms in order to tune its properties [3,4,5]. PCL is also used as a polymeric matrix to obtain nanocomposites by adding micro and nanoparticles to improve its mechanical properties and its thermal stability [6], thus taking also the advantage of the strong ability of PCL-based materials to be processed in different forms, such as bulk, films, and fibers, among others [7]. In particular, PCL fibers can be obtained by following different methods, such as melt-spinning, dry-spinning, and electrospinning, depending on the desired fiber properties as well as on the final applications [8,9,10].

The electrospinning process is the most suitable technique for drug delivery and tissue engineering applications of PCL-based nanocomposite fibers [11,12,13,14]. The electrospinning process, belonging to the electro-hydrodynamic processing [14], can consistently produce a wide variety of woven non-woven mat starting from polymeric solutions exposed to high electric fields that offer large surface areas, small inter-fibrous pore size, and high porosity [15,16,17,18], with interest in the biomedical field since they mimic the structure of extracellular matrix [19]. Moreover, the electrospinning process is one of the most efficient, simple, and versatile processing techniques able to control micro and nanofibers’ structural and functional properties through a relatively simple and cost-effective approach [20,21,22,23,24].

Furthermore, the development of nanocomposites is one of the most used approaches to improve PCL properties (i.e., thermal, mechanical, etc.) [6]. Among nanoparticles, cellulose derivatives have been considered optimal, reinforcing materials for biopolymers since they are bio-based, biodegradable, stiff, lightweight, and highly abundant in nature at low cost [22]. In particular, in the last years, cellulose nanocrystals (CNC), obtained from the acid hydrolysis of microcrystalline cellulose, have attracted the attention of many researchers [25,26]. However, CNC with high amounts of hydroxyl groups on their surface tends to aggregate, limiting improvements in the final properties of the nanocomposites [27,28,29]. Therefore, the homogeneous dispersion of high polarity CNC into the hydrophobic polymers matrices could be favored, modifying the nanocrystal surfaces by physical (i.e., use of surfactant) [30] or chemical modifications (*grafting*) [31]. The “grafting from” approach is particularly interesting since, in this method, different polymers can be directly grown in situ from the hydroxyl groups of the nanocellulose surface [31].

Chitosan is the second organic substance, most abundant in nature, after cellulose. Moreover, it has generated enormous interest due to its various advantages, such as easy availability, positive charge, renewability, biodegradability, biocompatibility, non-toxicity, and antimicrobial activity [32]. Another organic nanoparticle is graphene, which presents extraordinary mechanical properties, as high Young modulus and hardness and excellent flexibility are being considered as an effective reinforcement for high-performance nanocomposites [33,34].

On the other hand, PCL can be modified with inorganic nanoparticles, as hydroxyapatite, to improve its mechanical and biological properties for tissue engineering purposes. Hydroxyapatite is an inorganic component of bone with bioactivity and biocompatibility and shows good osteoconductivity and bone-bonding ability [35,36,37].

Finally, fumed silica is an inorganic and amorphous particle widely used at the industrial level mainly because it is a low-cost material obtained as a by-product of silicon [38]. Nanosized SiO_2_, in general, improves the mechanical performance of the corresponding nanocomposite, even at low concentration as a consequence of their small particle size [38,39]. Besides, metal nanoparticles have been extensively used as reinforcing agents to produce nanocomposites with several functional properties. Silver is one of the most conductive metals in nature, and silver nanoparticles (Ag) are known as a powerful antimicrobial agent, with an outstanding broad-spectrum of antibacterial effects against both Gram-positive and Gram-negative bacteria, and this is why Ag have been widely used in biomedical applications [40,41].

Therefore, in general, woven no-woven electrospun fiber mats can find applications in tissue engineering since they mimic the microarchitecture of the extracellular matrix [19,20]. Their structure can improve cell adhesion, proliferation, migration, and differentiation. Moreover, PCL electrospun mats can be used as a drug release system. Similar works are reported with cellulose nanocrystal and graphene [42,43]. Among other applications, silver nanoparticles are used as reinforcement for PCL-based scaffold for wound dressing due to their high antibacterial properties [44]. At the same time, scaffolds with fibrous structure can be designed for nerve tissue regeneration due to their morphological similarity of the organization of neurons by developing electrospun chitosan-PCL scaffold [45]. The incorporation of HA is supposed to produce an ideal scaffold suitable for bone tissue because it improves osteoblast proliferation and differentiation [46]. Graphene can be added not only to improve the mechanical properties of PCL but also to obtain electrospun nanofibers with high antibacterial activity [47,48]. At the same time, silica nanoparticles can be used as an additive to enhance the mechanical properties and/or trigger an osteogenic response from osteoblastic progenitor cells for guided bone regeneration used for the treatment of lesions in the alveolar or mandible bone [49].

In this work, the processing conditions to obtain PCL-based electrospun fibers reinforced with organic as well as inorganic nanoparticles were optimized. Firstly, neat PCL and PCL reinforced with 1 wt% of silica nanoparticles were used to optimize the processing parameters. The optimized processing conditions were then used to produce PCL-based electrospun nanocomposite fibers in order to study the effect of the addition of different nanoparticles on their thermal and mechanical responses. In particular, the nanofillers used as reinforcements were CNC, CNC-g-PCL, chitosan, and graphene as organic nanoparticles, while HA, Ag, and SiO_2_ were used as inorganic nanoparticles. All the nanoparticles were added to the PCL at 1 wt% in order to compare. The morphology, thermal behavior, and mechanical properties of the woven no-woven electrospun nanocomposite fibers were studied and compared with the neat PCL electrospun mat.

In particular, due to the intrinsic characteristic of both PCL polymer and the different nanoparticles, these electrospun nanocomposite mats could be considered for potential applications in bioactivity, such as antibacterial, biocompatibility, and biodegradability with proper mechanical performance.

## 2. Materials and Methods 

### 2.1. Materials

Poly(ε-caprolactone) (PCL) (PCL CAPA 6500, Mn = 50,000 g/mol, 0.5 wt% ε-caprolactone monomer) was kindly donated by Perstorp (Malmö, Sweden).

Chitosan (degree of deacetylation > 75%) [17], microcrystalline cellulose, as well as commercial hydroxyapatite nanofillers (HA, the particle size of about 30 nm), were purchased from Sigma-Aldrich (Madrid, Spain). Silver nanoparticles, Ag, (P203, Cima NanoTech, Caesarea, Israel) were previously purified by a thermal treatment, obtaining a specific surface area of 4.9 m^2^/g and particle size distribution from 20 to 70 nm [50]. Graphene nanoplatelets were supplied by Cheap Tubes Inc., Grafton, VT, USA (Grade 2) [51]. As indicated, they had a surface area of about 100 m^2^/g and an average thickness of a bit less than 10 nm. They were used as supplied by the manufacturer without any functionalization process. Fumed silica dioxide nanopowder, SiO_2_ (primary particle average size: 7–14 nm), was purchased from Interchim Innovations (Montluçon, France) [39]. Chloroform (99.6% purity) (CF) and dimethylformamide (DMF) (99.5% purity) were supplied by Sigma Aldrich (Madrid, Spain).

### 2.2. Synthesis of Cellulose Nanocrystals

Cellulose nanocrystals were obtained by sulfuric acid hydrolysis of 64% (wt/wt) of microcrystalline cellulose (MCC) stirring at 45 °C for 30 min [26,31]. The acid was further eliminated by centrifugation; the sediment was then dialyzed until neutral pH. An ion exchange resin was added to the cellulose suspension for 24 h, and it was then removed by filtration followed by ultrasonic treatment. Cellulose nanocrystal (CNC) solutions were then neutralized (1.0% (w/w) of 0.25 mol L^−1^ NaOH). Finally, the CNC solution was sonicated to get a stable suspension of the nanofillers.

### 2.3. CNC Functionalization with PCL Chains

**Scheme 1:**
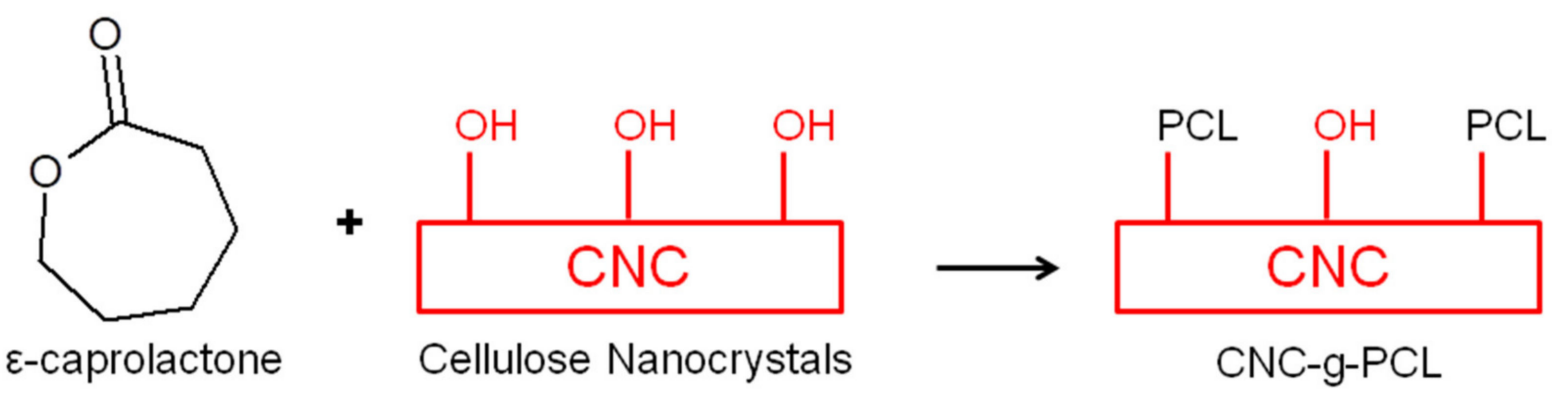
CNC with grafted PCL chains. CNC, cellulose nanocrystal; PCL, poly(e-caprolactone).

CNC surface chemical modification was performed by grafting PCL chains onto the CNC surface by ring-opening polymerization (*ROP*) of ε-caprolactone (ε-CL) by using the surface hydroxyl groups of the CNC as initiator, as schematically shown in Scheme 1. The procedure for CNC functionalization was previously reported for PCL grafting [28,31]. Briefly, the aqueous suspension of CNC was solvent-exchanged with acetone, then with dichloromethane, and finally with previously dried toluene with phosphorus pentoxide. For each solvent exchange step, the solution was centrifuged and re-dispersed three times.

The CNC-g-PCL nanocrystals were observed by field emission scanning electron microscopy, FESEM, (Hitachi S8000). The structure of CNC-*g*-PCL was characterized by Fourier-transform infrared (FT–IR) in the attenuated total reflectance (ATR) mode (as well as Raman spectroscopy), and its thermal stability was studied by thermogravimetric analysis (TGA), as indicated in the characterization section.

### 2.4. Preparation of Electrospun Nanofibers

Electrospun fibers were prepared by means of a coaxial Electrospinner Yflow 2.2.D-XXX (Nanotechnology Solutions) with a vertical standard configuration equipped with two concentric needles and connected to a high voltage power. The polymer solution flew through the inner needle, and the solvent flew through the outer one.

PCL electrospun fibers, named ePCL, were prepared from polymer solutions of PCL (10 wt%) in a solvent mixture of CHCl_3_:DMF (4:1) using a magnetic stirrer for 24 h at room temperature. The same solution was used to obtain PCL-based nanocomposite electrospun fibers by adding 1 wt% with respect to the polymer matrix of organic as well as inorganic nanoparticles, such as CNC, CNC-*g*-PCL, chitosan, graphene, Ag, HA, SiO_2_.

To prepare electrospun nanocomposite fibers, nanoparticles were dispersed separately (1 wt%) in the same mix of solvents using a magnetic stirrer for 2 h and ultrasonication for 2 min. Finally, to achieve homogeneous dispersion of the nanoparticles in the dissolved matrix, the polymer solution and nanoparticles’ suspensions were mixed, stirred (2 h), and sonicated (2 min) to form nanocomposite solutions with 1 wt% with respect to the matrix in the final composition.

The polymer solutions were flown through the inner needle, and the same mixture of solvent used for polymer solutions was flown through the outer needle. The applied positive and negative voltages were set at 10 and −10 kV, respectively. The polymer flow rate, as well as the solvent flow rate, were fixed at 1.0 mL/h. Electrospun mats were randomly collected in a grounded aluminum foil collector situated perpendicular at 15 cm from the charged spinneret. The obtained electrospun mats were vacuumed for 48 h in a vacuum chamber to eliminate residual solvents before testing.

### 2.5. Characterization Techniques

#### 2.5.1. Vibrational Spectroscopy: Raman and FT-IR Spectra

Neat CNC and functionalized CNC were characterized by Raman spectra using a RenishawInVia Reflex Raman system. An optical microscope was coupled to the system. The Raman scattering was excited using a diode laser at a wavelength of 785 nm. The laser beam was focused on the sample with a 100 × 0.85 microscope objective. The laser power at the sample was 350 mW. The exposure was for 10 s, and there were 10 accumulations for the Raman measurements.

Fourier-transform infrared (FT–IR) spectra were obtained in the attenuated total reflectance (ATR) mode. The measurements were performed using a Spectrum One FTIR spectrometer Perkin Elmer equipped with an internal reflection element of diamond in the range of 650–4000 cm^−1^ with 1 cm^−1^ of resolution and an accumulation of 16 scans.

#### 2.5.2. Scanning Electron Microscopy

The morphology of PCL-based electrospun nanocomposite fibers was analyzed by means of scanning electron microscopy (SEM). Thus, electrospun fibers were sputtered with a gold/palladium layer and observed by a PHILIPS XL30 scanning electron microscope (SEM). Fiber diameters were statistically calculated by means of Fib_thick software executable under image analysis platform Fiji based on ImageJ.

#### 2.5.3. Thermal Analysis

TGA analysis was performed to study the thermal degradation of the electrospun samples. Thermogravimetric analysis (TGA) was performed in a TA-TGA Q500 thermal analyzer. Dynamic TGA experiments were performed under a nitrogen atmosphere (flow rate of 60 mL/min). Samples were heated from room temperature to 700 °C at 10 °C·min^−1^. In this case, the maximum degradation temperature (T_max_) was calculated from the first derivative of the TGA curves.

The thermal behavior, as well as the degree of crystallinity (X_c_), were studied by differential scanning calorimetry (DSC). DSC experiments were conducted in a Mettler Toledo DSC822 instrument under a nitrogen atmosphere (50 mL/min). Sample weights of about 4 mg were sealed in aluminum pans and heated from −90 to 100 °C at a heating rate of 10 °C min^−1^. The glass transition temperature (T_g_) was taken at the midpoint of heat capacity changes. The melting temperature (T_m_) and cold crystallization temperature (T_cc_) were obtained from the first heating, and the degree of crystallinity (χc) was calculated through Equation (1):

(1) $$\chi_{c}=100\%\times \left[\frac{\Delta H_{m}-\Delta H_{cc}}{\Delta H_{m}^{c}}\right]$$

where Δ*H_m_* is the melting enthalpy, Δ*H_cc_* is the cold crystallization enthalpy, and $\Delta H_{m}^{c}$ is the melting heat associated with pure crystalline PCL (148 J g^−1^) [5].

#### 2.5.4. Mechanical Analysis

The mechanical properties of the PCL-based electrospun nanocomposite mats were investigated by tensile test measurements determined at room temperature in an Instron dynamometer (model 3366) equipped with a 100 N load cell, at a crosshead speed of 10 mm min^−1^ and initial length of 30 mm. Dog-bone samples were prepared from the mats, and, at least, five specimens were tested for each formulation. From these experiments, the Young Modulus—as the slope of the curve between 0% and 2% of deformation—the elongation at break, and the maximum strain reached were obtained.

## 3. Results

### 3.1. CNC Synthesis and Functionalization

First, the synthesis and the characterization of the different organic and inorganic nanoparticles were performed. In particular, neat cellulose nanocrystals (CNC) were synthesized and functionalized in our lab, while the other nanoparticles used in this work were purchased. 

In particular, cellulose nanocrystals were synthesized in the laboratory by acid hydrolysis of commercial microcrystalline cellulose. Then, surface modification of the CNC was carried out by a “grafting from” reaction, grafting PCL chains onto the CNC surface by ring-opening polymerization (ROP) of –CL, using the surface hydroxyl groups of the CNC as initiator [28].

**Figure 1:**
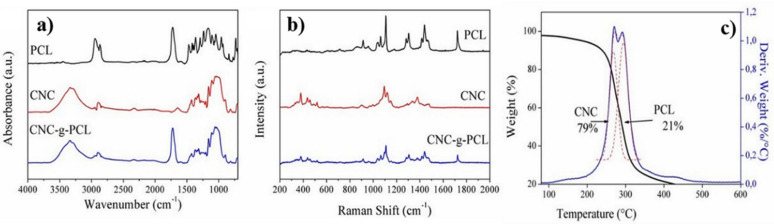
Vibrational spectroscopy: (**a**) FTIR spectra for PCL, CNC and CNC-*g*-PCL, (**b**) Raman spectra for PCL, CNC and CNC-*g*-PCL, and (**c**) thermogravimetric analysis of CNC-*g*-PCL. CNC-*g*-PCL: CNC with grafted PCL chains.

In particular, in order to ensure the presence of PCL chains in CNC-*g*-PCL during the “grafting from” reaction, vibrational spectroscopies studies were conducted, comparing the results obtained from neat PCL and CNC with CNC grafted with PCL (Figure 1). The FTIR spectrum of the CNC-*g*-PCL (Figure 1a) showed the characteristic peaks of CNC and of PCL. It was possible to observe a broad peak corresponding to the hydroxyl groups of CNC in the 3200 cm^−1^ to 3600 cm^−1^ region and a shaper peak of carboxyl groups of PCL at 1721 cm^−1^, confirming the presence of both of them in the nanoparticles. Figure 1b shows the Raman spectrum of the PCL, CNC, and CNC-*g*-PCL, confirming the presence of PCL and CNC in the sample. These results confirmed the success of the grafting procedure.

Moreover, TGA was used to determine the amount of CNC and PCL in the CNC-g-PCL nanoparticles. Figure 1c shows the weight loss and the derivative of weight loss for CNC-*g*-PCL. Two overlapped peaks were observed, in which one of them corresponded to the thermal degradation of CNC and the other one to the thermal degradation of PCL chains. CNC peak presented a maximum corresponding to 270 °C, and PCL showed the maximum peak at 292 °C. The amounts of the components were computed by fitting the curves with two Gaussian curves, so the amount of PCL grafted chains resulted as 21 wt%, while the amount of CNC was 79 wt%, in the range of already reported results [28].

### 3.2. Morphological Analysis

**Figure 2:**
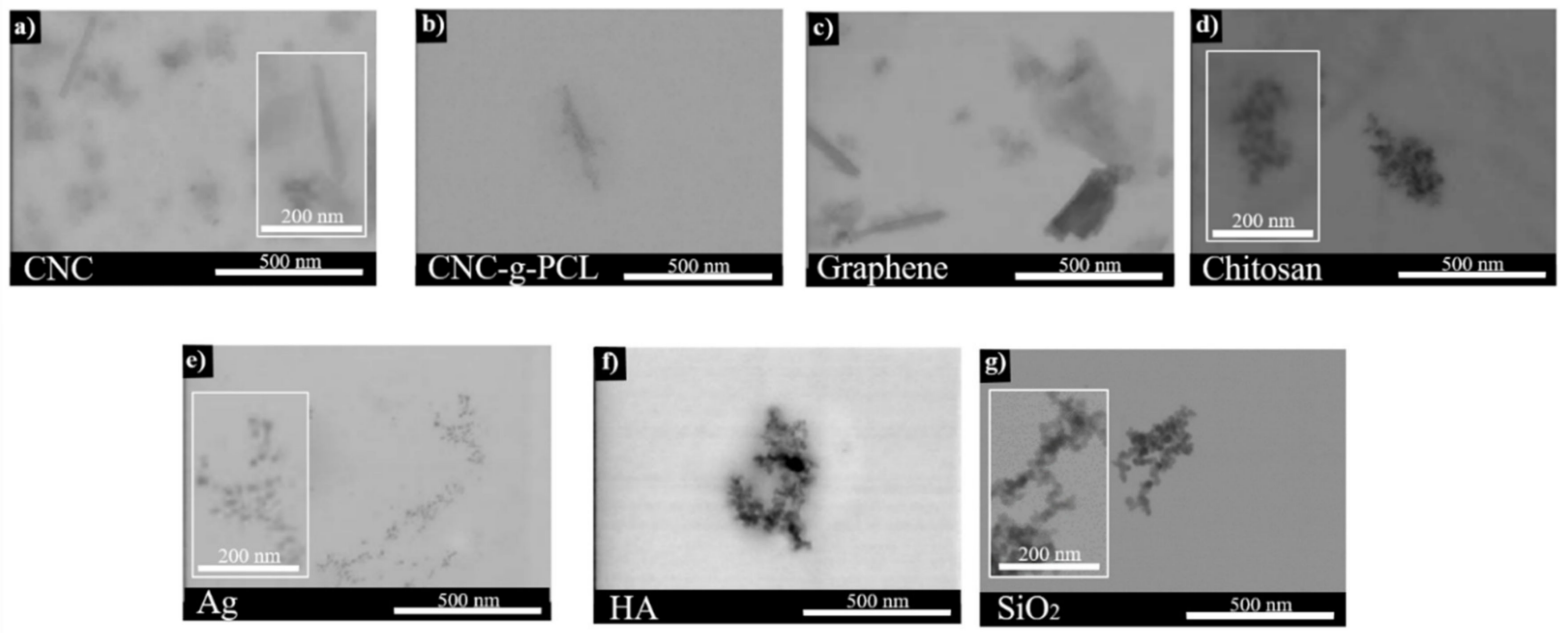
FE-SEM images of the different nanoparticles used in this work: (**a**) CNC, (**b**) CNC-*g*-PCL, (**c**) Graphene, (**d**) Chitosan, (**e**) silver (Ag), (**f**) hydroxyapatite (HA), (**g**) silica (*SiO_2_*) nanoparticles.

Morphological analysis was performed for all the organic and inorganic nanoparticles used and reported in Figure 2.

From the FE-SEM images, the nanometer range size of the different nanoparticles was confirmed. In particular, the average length of CNC was determined by image analysis (ImageJ software), obtaining an average value of 179 ± 20 nm for the length and of 14.1 ± 1.6 nm for the diameter (Figure 2a), in good agreement with previous synthesized CNC [26]. Figure 2b shows individual nanoparticles of CNC-*g*-PCL, showing the characteristic rod-like morphology. The average length of CNC-*g*-PCL was determined by image analysis (ImageJ software), obtaining a value of 317 ± 59 nm for the length and a value of 24.4 ± 10.0 nm for the diameter, respectively, indicating an increase in both length and diameters due to the polymeric chain grafted onto the surface of the cellulose nanocrystals, in accordance with previous works [28].

Regarding the other nanoparticles used in the present work, the average dimension measured was in accordance with the values indicated by the seller, as indicated in Table 1. However, we could summarize that all the nanoparticles used in this work were smaller than 50 nanometers and could be easily used in the electrospinning process (Figure 2).

### 3.3. Optimization of the Electrospinning Process Parameters

Once the nanoparticles were characterized, we proceeded with the optimization of the electrospinning process and the characterization of the woven no-woven reinforced and no-reinforced PCL-based electrospun fiber mats. In particular, for the electrospinning process, PCL should be homogeneously dissolved in a proper solvent, and it is known that an effective solvent should present a similar solubility parameter (δ) to that of the polymer [52]. The solubility parameter of PCL is between 15.9 and 21.2 MPa^1/2^ [53]. Chloroform (CF) has shown to be an effective solvent for PCL; meanwhile, dimethylformamide (DMF) is normally used to facilitate the electrospinning process. CF and DMF have solubility parameters of 19 MPa^1/2^ and 24.9 MPa^1/2^, respectively [15]. Accordingly, good solubility in CF and DMF for PCL should be expected.

**Figure 3:**
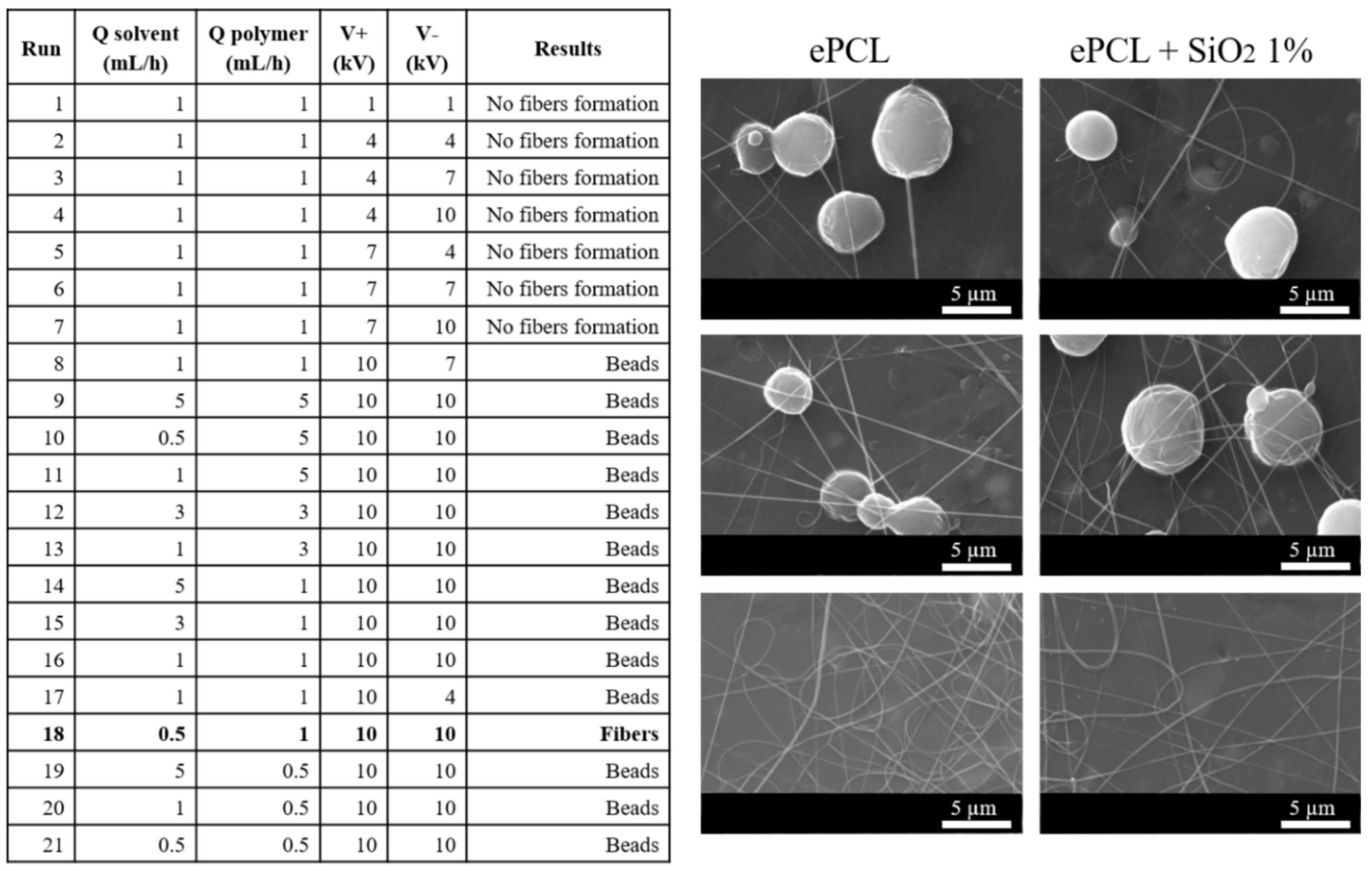
Optimization of the electrospinning process: processing-window parameters and SEM images for PCL and PCL reinforced with silica nanoparticles.

The electrospinning processing conditions used to prepare electrospun fibers were selected, taking into account the optimization carried out for the production of neat PCL electrospun fibers (ePCL), as well as for silica-reinforced PCL electrospun fibers (ePCL + SiO_2_). Both neat PCL electrospun fibers and PCL-based electrospun nanocomposite fibers were obtained from a PCL solution (10 wt%) and nanoparticle dispersions (1 wt%) in a mixture of solvents (chloroform-DMF 4:1). Firstly, the PCL solution was prepared, and, at the same time, nanofillers were dispersed separately. Then, the polymer solution and nanoparticles’ suspensions were mixed, and then the final stable dispersion of nanoparticles into the PCL solution was obtained after sonication. Thus, the concentration of PCL solutions was set at 10 wt%, and the working distance between the needle and the collector was set at 15 cm [15,22]. The solvent and polymer flow rate, as well as the positive and negative voltage, were optimized according to the experimental results reported in Figure 3.

According to the fiber formations for both neat PCL and PCL-based electrospun nanocomposite fibers, reported in the table of Figure 3, it was worth noting that when small voltages were applied, the fiber formation was avoided. Both neat PCL and PCL-based electrospun fibers were formed upon increasing the voltage applied. However, for high flow rates of the polymer solution, typical defects, named beads, were obtained (see Figure 3). The best conditions for both neat and reinforced electrospun fibers were with a small flow rate in the solvent solution pump, a flow rate of 1 mL/h for the polymer solutions, and a high voltage (run 18). In particular, the electrospun fibers obtained with these conditions are reported in Figure 3 for both ePCL and ePCL reinforced with silica nanoparticles. These results showed that to ensure the formation of the so-called Taylor cone, and hence the formation of electrospun fibers without defects, higher Coulomb forces were required to favor the elongation of the polymeric drops, and thus, higher voltage values were required. Therefore, we considered these conditions as optimum to obtain and to compare the properties of neat PCL as well as of PCL-based electrospun fibers reinforced with both organic and inorganic nanoparticles at 1 wt%.

In accordance with the literature, we chose to study 1 wt% of different organic and inorganic nanoparticles, considering that this amount of nanoparticles could be effectively dispersed into the CHCl_3_:DMF (4:1) solution, while higher concentrations produce a detriment of the structural and mechanical performance due to their reduced dispersion [22]. It was quite difficult to fix a unique amount of very disparate types of nanoparticles in order to compare the final properties of the nanocomposite electrospun fibers. Therefore, when we referred to the literature, in the case of CNC, we found that 1 wt% is a good amount to be dispersed into electrospun fibers [54]. Low amounts are also used for chitosan (i.e., 1 wt%) [17]. At the same time, Correa et al. developed electrospun scaffolds based on PCL reinforced with reduced graphite oxide (rGO) at concentrations up to 1 wt% [54]. Inorganic nanoparticles can be used also at low content. In fact, Ribeiro Nieto et al. studied PLA and PCL electrospun fibers reinforced with 1 wt% and 5 wt% of nano-sized hydroxyapatite, and the highest Young modulus was found for bionanocomposites reinforced with 1 wt% HA, also showing viable cells with early osteogenic activity [8]. Therefore, in this work, 1 wt% was the amount of both organic and inorganic nanoparticles added to the PCL electrospun mats.

**Figure 4:**
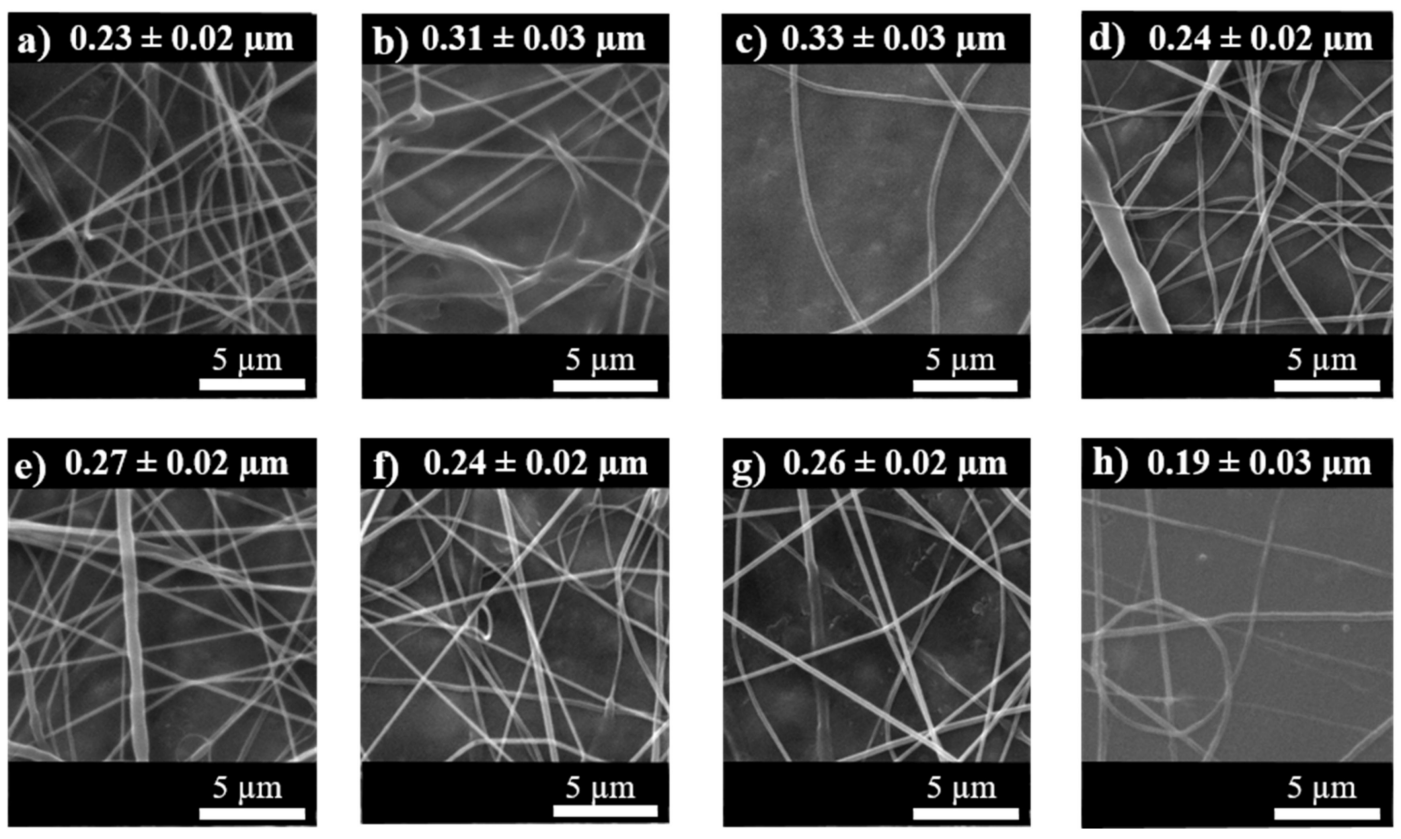
SEM images of (**a**) ePCL, (**b**) ePCL/CNC, (**c**) ePCL/CNC-g-PCL, (**d**) ePCL/Graphene, (**e**) ePCL/Chitosan, (**f**) ePCL/Ag, (**g**) ePCL/HA, (**h**) ePCL/SiO_2_ electrospun fibers as well as their corresponding average diameters.

Therefore, the electrospinning process was realized for all the systems, and the ePCL, as well as the PCL-based electrospun fiber mats, were obtained using the same processing window optimized before. Thus, the electrospun fibers based on PCL and reinforced with the organic, such as CNC and CNC-g-PCL, chitosan and graphene, as well as the other inorganic nanoparticles, such as silver and hydroxyapatite, were obtained. SEM images for all the PCL-based electrospun fiber mats are reported in Figure 4, and the corresponding average fiber diameters—for each system studied—are indicated on the top of each image.

Thermal and mechanical characterization was also carried out. In particular, the main result of the thermal characterization is reported in Table 2, where the glass transition temperature (T_g_), the melting temperature (T_m_), and the degree of crystallinity (X_c_) calculated from the DSC analysis and the maximum degradation temperature from TGA analysis are reported.

### 3.4. Thermal Analysis

In particular, from the thermal point of view, no significant differences in the T_g_ and T_m_ of the PCL-based electrospun fibers, as well as in their degree of crystallinity, were emerged from their comparison (Table 2). However, the degree of crystallinity of ePCL was quite high, 52%, indicating that the balance between the fiber formation and the solvent evaporation produced a quite crystalline material. Moreover, the addition of the nanoparticles did not alter the high degree of crystallinity of the neat ePCL. Different behavior, for example, was noted when working with other polymers, such as poly(lactic acid), where its neat electrospun fibers show a very low degree of crystallinity [23].

On the other hand, when we considered the maximum degradation temperature, it was evidenced that all the nanoparticles increased the maximum degradation temperature of the ePCL. The highest value was obtained when functionalized CNC and chitosan were added to the PCL. It seemed that the smallest increment was obtained when inorganic nanoparticles were added to the ePCL, considering that the addition of SiO_2_ nanoparticles did not change the Tmax of neat ePCL, and the addition of both Ag and HA nanoparticles increased the Tmax of neat ePCL by about 1%.

For these reasons, we concluded that it was not possible to classify the different effects produced from the addition of organic and inorganic nanoparticles on the ePCL electrospun mats in terms of thermal characterization.

### 3.5. Mechanical Characterization

**Figure 5:**
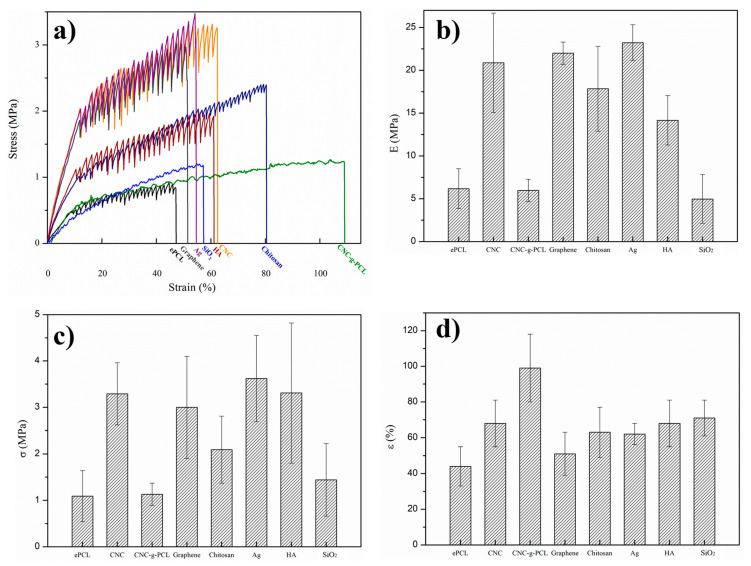
Mechanical response for the neat PCL and the reinforced PCL-based electrospun fibers. Stress-strain diagrams (**a**), Young modulus (**b**), tensile strength (**c**) and elongation at break (**d**) for all the samples studied.

The mechanical characterization of all the different electrospun nanocomposite systems based on PCL was also performed by tensile test measurements (Figure 5a). First of all, it was important to point out that the measurement of “electrospun mats” was quite different from PCL “bulk” materials, considering the presence of fiber entanglements and the many microvoids between the fiber webs, producing very high error measurements, as indicated by the large error bars in Figure 5. Moreover, it was worth noting that the mechanical response of ePCL was quite different from the PCL bulk material, thus considering that when PCL was obtained in the form of electrospun fibers, the values of about 45% for the deformation at break were obtained. However, the ePCL electrospun nanocomposite fibers showed their reinforcing effects with respect to the neat ePCL in terms of Young modulus, tensile strength, as well as elongation at break, as indicated in Figure 5b–d, respectively.

Analyzing the different systems studied, both organic and inorganic nanofillers were able to increase the Young modulus and the tensile strength of electrospun PCL mat. The smallest effects in terms of Yong modulus and tensile strength were presented when CNC-*g*-PCL and SiO_2_ were added to the PCL matrix; however, their elongation at break was strongly increased. In fact, the electrospun nanocomposite mat reinforced with CNC-*g*-PCL showed the highest elongation at break, with an increment of about 220% with respect to ePCL, suggesting that the better compatibilization between PCL and CNC was reached by grafting PCL chains onto the CNC surfaces. Regarding ePCL/SiO_2_, although it was not able to increase the mechanical resistance of ePCL, it was able to increase the flexibility of the material by increasing the elongation at break by about 155% with respect to ePCL.

However, all the electrospun nanocomposite fibers showed improved elongation at break, with increments higher than 150% with respect to ePCL.

Furthermore, also in the case of the mechanical response, it was quite impossible to classify the reinforcement effect depending on the use of organic or inorganic nanoparticles. In fact, the addition of Ag nanoparticles, graphene, and CNC to the ePCL electrospun fibers provided an increment of more than 350% in terms of Young modulus with respect to ePCL, while the electrospun PCL fibers reinforced with chitosan and with HA showed an increment of about 250% with respect to the Young modulus of ePCL.

For the tensile strength, as said before, the addition of both CNC-*g*-PCL and SiO_2_ nanoparticles slightly increased the tensile strength with respect to ePCL; however, the addition of CNC, graphene, Ag, and HA increased the tensile strength by about 300%.

**Figure 6:**
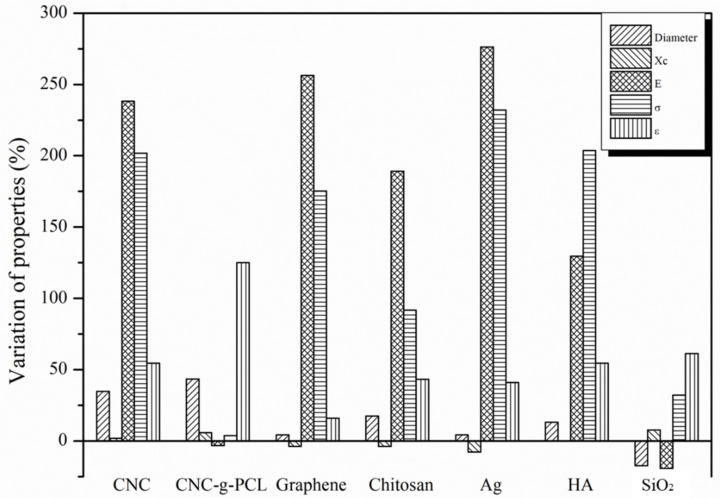
Variation of the properties of the woven no-woven electrospun nanocomposite systems with respect to the ePCL values.

Finally, in Figure 6, the variation of the properties of the woven no-woven electrospun nanocomposite systems with respect to the ePCL values has been summarized in order to better visualize the effect of the addition of the different organic and inorganic nanoparticles on the fiber diameter, degree of crystallinity, and the mechanical response.

## 4. Discussion

Summarizing, the values for the degree of crystallinity were quite similar, hindering a clear separation on the effect of the addition of organic or inorganic nanoparticles being strongly influenced by the high crystallinity of the neat ePCL electrospun fiber mats—higher than 50%.

Firstly, on comparing the properties of woven no-woven electrospun fibers and their corresponding bulk materials, we found that they were completely different. This was the case of PCL, but when we considered other polymers, such as PLA, the electrospun fiber mats presented a completely different mechanical response from bulk. For instance, PLA in bulk presents a Young modulus much higher and an elongation at break very smaller than PLA electrospun mats [55]. For PCL electrospun materials, the effect was different with respect to PLA-based electrospun mats. In fact, in our previous work [56], we reported the mechanical response of the same PCL obtained by the extrusion process, obtaining an elastic modulus of 294 ± 18 MPa, the maximum stress of 41 ± 4 MPa, and an elongation at break of 952 ± 23%. All the values obtained in bulk were much higher than the mechanical response obtained in electrospun fiber forms, where the elastic modulus obtained was about 6 MPa, the tensile strength was about 1 MPa, and the elongation at break was less than 50%. It was clear that PCL in the form of woven no-woven electrospun mats lost its capability of being a very flexible material.

It is expected that the elastic modulus and the tensile strength of the electrospun PCL-based fibers increase as the fiber diameter decreases [57]. Nevertheless, it could be observed that in the case of organic PCL-based electrospun fibers, the flexibility of the material increased with increasing the average fiber diameter. This behavior was particularly evident in the case of functionalized CNC (CNC-*g*-PCL), which showed a higher average fiber diameter and the higher elongation at break. Considering the inorganic nanofillers, it was observed that SiO_2_ nanoparticles were able to produce the highest increment on the elongation at break among inorganic PCL-based electrospun fibers. However, it was quite difficult to differentiate the mechanical response of the PCL-based electrospun nanocomposite fibers regarding the effects of organic and inorganic nanofillers as a clear trend was not found.

It is well known that the different morphologies of the nanoparticles (0 D, 1 D, or 2 D dimensions) can strongly affect the mechanical response as well as the degree of crystallinity of the electrospun fibers [6]. However, in our case, it was difficult to differentiate the main results in terms of thermal or mechanical properties, depending on the geometry of the nanoparticles. In fact, the highest Young modulus and tensile strength were obtained with CNC, rod-like shaped Ag with spherical geometry, and graphene with a layered structure, indicating that the three different geometries provided the highest mechanical response.

## 5. Conclusions

Woven no-woven electrospun fibers based on PCL and reinforced with both organic and inorganic nanoparticles were obtained. As organic nanoparticles, we used cellulose nanocrystals, chitosan, and graphene, while, as inorganic nanoparticles, we used silver, hydroxyapatite, and fumed silica nanoparticles. In particular, cellulose nanocrystals (CNCs) were synthesized and functionalized by “*grafting from*” reaction, using their superficial OH– group to graft PCL chains. All the nanoparticles were added at 1 wt% with respect to the polymeric matrix, in order to be compared to each other. No difference was found between the effect of the addition of organic or inorganic nanoparticles on the thermal properties, considering that no significant variation in the T_g_, T_m_, and degree of crystallinity was obtained, leading to a high crystallinity electrospun mats with a degree of crystallinity of the electrospun PCL matrix of about 50%. However, all the nanoparticles increased the maximum degradation temperature of the respective nanocomposite electrospun fibers, indicating a good interaction between nanoparticles and the polymeric matrix. From the mechanical point of view, the highest values of Young modulus were obtained when graphene, CNC, and silver nanoparticles were added to the ePCL. On the other hand, all the nanoparticles used, both organic and inorganic types, increased the flexibility of the electrospun mats, increasing the elongation at break.

## Figures and Tables

**Table 1:** Nanoparticles used and their dimensions.

Nanoparticles	Dimensions
CNC	179 ± 20 nm 14.1 ± 1.6 nm
CNC-*g*-PCL	317 ± 59 nm 24.4 ± 10.0 nm
Graphene	286.6 ± 64.6 nm 35.5 ± 1.0 nm
Chitosan	23.7 ± 2.3 nm
Ag	22.4 ± 2.4 nm
HA	18.8 ± 2 nm
SiO_2_	24.5 ± 2.9 nm

CNC: Cellulose nanocrystals; CNC-g-PCL: CNC with grafted PCL chains; *HA*: hydroxyapatite.

**Table 2:** Thermal characterization for the neat PCL and the reinforced PCL-based electrospun fibers.

Samples	T_g_ (°C)	T_m_ (°C)	X_c_ (%)	T_max_ (°C)
ePCL	−63	63	52	398
ePCL/CNC	−65	65	53	405
ePCL/CNC-g-PCL	−60	61	55	417
ePCL/Graphene	−62	62	50	406
ePCL/Chitosan	−62	64	50	417
ePCL/Ag	−62	64	48	400
ePCL/HA	−62	61	52	402
ePCL/SiO_2_	−65	63	52	398

Electruspun PCL fibers (ePCL), ePCL reinforced with cellulose nanocrystals (ePCL/CNC), ePCL reinforced with CNC grafted with PCL chains (ePCL/CNC-g-PCL), ePCL reinforced with silver nanoparticles (ePCL/Ag), ePCL reinforced with hydroxyapatite (ePCL/HA), ePCL reinforced with fumed silica nanoparticles (ePCL/SiO2).
